# Detection of *Salmonella* species in chicken carcasses using genus specific primer belong to *invA* gene in Sohag city, Egypt

**DOI:** 10.14202/vetworld.2016.1125-1128

**Published:** 2016-10-22

**Authors:** Nahed Mahmoud Abdel-Aziz

**Affiliations:** Department of Food Hygiene, Faculty of Veterinary Medicine, Sohag University, Naser Street, Sohag, Egypt

**Keywords:** chicken carcass, *invA* gene, *Salmonella* spp

## Abstract

**Aim:**

This study aimed to detect *Salmonella* species found as contaminants in chicken carcass (thigh, breast, wings, liver, and gizzard).

**Materials and Methods:**

A total of 75 chicken samples including thigh, breast, wings, liver, and gizzard (15 of each) were collected from different markets in Sohag city for detection of *Salmonella* species by culture methods, biochemical tests, serology, and polymerase chain reaction.

**Results:**

The overall incidence of *Salmonella* contamination of 75 examined samples was found to be 6.6% with the higher percentage of *Salmonella* being isolated from liver samples (13.3%) followed by thigh, wings, gizzard (6.6%) while breast show negative result.

**Conclusion:**

Results in this study indicate that contamination of chicken carcass with *Salmonella* needs strict hygienic measures to prevent their transmission to human.

## Introduction

Poultry meat constitutes a substantial portion of protein in this day diets; hence, it has an important share (30%) in the world’s total meat consumption [1]. The poultry meat is easy to prepare at home and widely used in restaurants and fast-food establishments [2]. Poultry products have always topped the incidence of salmonellosis in many developing countries including India, Egypt, Brazil, and Zimbabwe [3]. *Salmonella* often reach the carcasses from the intestinal tracts or fecal materials on feathers or feet. Particularly scalding, defeathering, evisceration, and giblet operations are the major points of spread in poultry processing plants [4].

There are several transmission routes for salmonellosis, but the majority of human infections are derived from the consumption of contaminated foods especially those of animal origin [5]. In human, *Salmonella* is the cause of two diseases called Salmonellosis: Enteric fever (typhoid), resulting from bacterial invasion of the blood stream, and acute gastroenteritis, resulting from a foodborne infection/intoxication [6].

Polymerase chain reaction (PCR) technology is used for rapid detection [7] and increase the sensitivity of detection of *Salmonella* in food, environmental, and clinical samples. The *invA* gene is the target of many of these methods as it is found in all known serovars of *Salmonella* [8]. Furthermore, it codes for protein in the inner membrane of bacteria that are necessary for invasion of epithelial cells [9]. This study aimed to detect *Salmonella* spp. from poultry products using traditional detection methods as culturing, biochemical tests, serology, and PCR detection of *invA* gene.

## Material and Methods

### Ethical approval

Not required for this study.

### Collection of samples

A total of 75 samples were collected from different markets in Sohag city. The collected samples include thigh, breast, wings, liver, and gizzard (15 of each). The samples were transported immediately to the laboratory in the Faculty of Veterinary Medicine in Sohag University.

### Isolation of *Salmonella* species

About 25 g of each sample was cut into small pieces using sterile forceps and scissors and blended for 2 min in sterile blender jar containing 225 ml of buffered peptone water (0.1%) as a pre-enrichment broth and incubated at 37°C for 24 h. After incubation, 0.1 ml of pre-enrichment culture was transferred into sterile tubes containing 10 ml of Rappaport Vassiliadis broth (Lab M Ltd., UK), and the tubes were then incubated at 43°C for 24 h. Thereafter, a loopful of each incubated tube was cultured on xylose lysine desoxycholate (Hi Media, India) agar plates and incubated for 24 h at 35°C. Typical colony of *salmonella* appears as pink colonies with or without black centers.

### Identification of *Salmonella* spp.

Identification was done morphologically by microscopical examination with Gram-stain.

#### Biochemical identification

Biochemical tests as triple sugar iron (Oxoid, UK) reaction, urease test (Oxoid, UK), indole production, methyl red (Becton Dickinson) test, and simmons citrate (Titan media, India) test. Isolates proved biochemically to be *Salmonella* spp.

#### Sereological identification

Serological identification was done according to Kauffmann-White scheme [10] in the Food Hygiene Lab in the Faculty of Veterinary Medicine, Banha University, Egypt.

#### Detection of invA gene using PCR

Detection of the invasion gene (invA) was performed according to the primer sequence 5’GTGAAATTATCGCCACGTTCGGGCA’3 and 5′TCATCGCACCGTCAAAGGAACC’3 according to Shanmugasamy *et al*. [11]. The isolated colonies from samples were overnight cultured on nutrient agar (Oxoid) plates, one or two colonies were suspended in 20 ml of sterile distilled water, and the suspension was then heated at 100°C for 20 min. Accurately, 50-200 µl of the culture was placed in Eppendorf tube and the DNA extraction occurred using QIA amp kit [12]. The amplification was performed on a thermal cycler (Mastercycler, Eppendorf, Hamburg, Germany). The PCR cycling protocol was applied as following: An initial denaturation at 94°C for 5 min, followed by 35 cycles of denaturation at 94°C for 60 s, annealing at 64°C for 30 s, and extension at 72°C for 30 s, followed by a final extension at 72°C for 7 min. Finally, 5 µl of each amplicon was electrophoresed in 1.5% agarose gel (Sigma, USA) and visualized under ultraviolet transilluminator. A 100 bp DNA ladder was used as a marker (Promega, USA) for PCR products.

## Results and Discussion

*Salmonella* species is present in the examined chicken carcasses with percentage of 6.6%, the highest level of contamination was present in liver with percentage of 13.3 followed by thigh, wings and gizzard 6.6%, while breast samples free from *Salmonella* species. Owing to *Salmonella* serotypes, the rate of contamination with *Salmonella enteritidis* and *Salmonella typhimurium* was 2.7% and *Salmonella Kentucky* 1.3%. The *invA* gene was used for specific identification of *Salmonella* species using PCR.

Meat and poultry products are recognized as the major sources for transmitting *Salmonella* species to human with 40% of the clinical cases attributed to the consumption of egg and poultry products [13]. In this study, the overall incidence of *Salmonella* contamination of examined samples was found to be 6.6% (Table-1). These results closely agree with Akbar and Kumar [14], Karmi [15], Suwit *et al*. [16], and Pedro *et al*. [17]. Compared with other studies that evaluated chicken carcasses, the prevalence of *Salmonella* spp. In this study was lower than that recorded by Alali *et al*. [18], Jianghui *et al*. [19], Rodriguez *et al*. [20], and Jarquin *et al*. [21], whereas this result was higher than that obtained by Elgroud *et al*. [22]. On the opposite side, Cretu *et al*. [23] reported that some countries such as Sweden where poultry free from *Salmonella*, and this stage was reached after observing some governmental control programs and measures, applied by poultry breeders and meat processors.

**Table-1:** Incidence of *Salmonella* contamination of the examined samples.

Samples	Number of the examined samples	Number of the isolates	%
Thigh	15	1	6.6
Breast	15	0	0
Wings	15	1	6.6
Liver	15	2	13.3
Gizzard	15	1	6.6
Total	75	5	6.6

Currently, *Salmonella* is detected by standard bacteriological, biochemical, and serological techniques. These techniques are generally time-consuming, tedious, and expensive [24]. *Salmonella* specific PCR with primers for *invA* gene is rapid, sensitive, and specific for detection of *Salmonella* in many clinical samples. The *invA* gene is carried on a region of the bacterial chromosome known as the *Salmonella* pathogenicity island 1 and encodes for protein in inner membrane of bacteria, which is necessary for invasion to epithelial cells for full virulence in *Salmonella* and is thought to trigger internalization required for invasion of deeper tissue [25].

**Figure-1:**
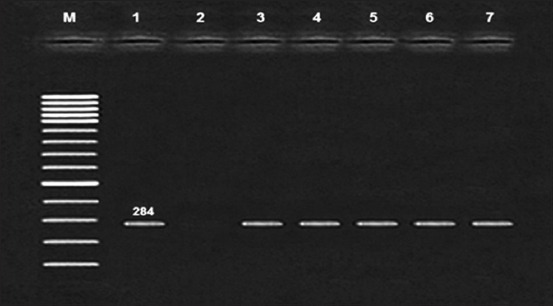
Agarose gel electrophoresis of polymerase chain reaction of *invA* gene (284 bp) for identification and characterization of *Salmonella* species. Lane M: 100 bp ladder as molecular size DNA marker. Lane 1: Control positive *Salmonellae invA* gene, Lane 2: Control negative, Lanes 3, Lane 4, Lane 5, Lane 6 and Lane 7: Positive *Salmonellae invA* gene.

Serological tests in this study revealed that the five isolates belonged to three different serotypes, *Salmonella typhimurium* 2 (2.7%)*, Salmonella enteritidis* 2 (2.7%), and *Salmonella kentucky* 1 (1.3%) (Table-2). The results of serological identification of *Salmonella* species in this study improve the result obtained by Ibrahim *et al*. [26] who found that the most common serotypes in carcasses surveyed were the main serotypes of *Salmonella* found in the literature associated with disease in humans, *Salmonella enteritidis* and *typhimurium*. *Salmonella enterica* serovar, *Salmonella enteritidis*, and *Salmonella* *enterica* serovar *typhimurium* are the most frequently isolated serovars from foodborne outbreaks worldwide [27]. Figure-1 showed confirmatory identification of *Salmonella* species by PCR method with genus specific primer called *invA* gene, using this method is accurate, rapid and less expensive. In conclusion, isolation of *Salmonella* carrying invasion *invA* gene in this study may indicate the poor sanitation of the environment under which chicken is slaughtered and increases the burden of foodborne infections in the people, and emphasizes the need to continuous education of the consumers on proper food handling and cooking practices to decrease the risk of transmission of *Salmonella* and other foodborne pathogens.

**Table-2:** Serological identification of Salmonellae species.

*Salmonella* serovar	N=75 (%)	Sample	Group	Antigenic structure	

				O	H
*Salmonella enteritidis*	2 (2.7)	Thigh, liver	D1	1, 9, 12	g, m
*Salmonella typhimurium*	2 (2.7)	Wings, gizzard	B	1, 4, 5, 12	i: 1, 2
*Salmonella kentucky*	1 (1.3)	Liver	C3	8, 20	i: Z6

## Authors’ Contributions

NMA designed the study, collected and analyzed the samples, drafted and revised the manuscript.
